# Unveiling the Essential Role of Arkadia’s Non-RING Elements in the Ubiquitination Process

**DOI:** 10.3390/ijms231810585

**Published:** 2022-09-13

**Authors:** Maria Birkou, Georgia N. Delegkou, Konstantinos D. Marousis, Nefeli Fragkaki, Tamara Toro, Vasso Episkopou, Georgios A. Spyroulias

**Affiliations:** 1 Department of Pharmacy, University of Patras, 26504 Patras, Greece; 2 Department of Brain Sciences, Imperial College, London W12 0NN, UK

**Keywords:** non-RING elements, E3 ligases, E2 enzymes, Arkadia

## Abstract

Arkadia is a positive regulator of the TGF*β*-SMAD2/3 pathway, acting through its C-terminal RING-H2 domain and targeting for degradation of its negative regulators. Here we explore the role of regions outside the RING domain (non-RING elements) of Arkadia on the E2-E3 interaction. The contribution of the non-RING elements was addressed using Arkadia RING 68 aa and Arkadia 119 aa polypeptides. The highly conserved NRGA (asparagine-arginine-glycine-alanine) and TIER (threonine-isoleucine-glutamine-arginine) motifs within the 119 aa Arkadia polypeptide, have been shown to be required for pSMAD2/3 substrate recognition and ubiquitination in vivo. However, the role of the NRGA and TIER motifs in the enzymatic activity of Arkadia has not been addressed. Here, nuclear magnetic resonance interaction studies with the E2 enzyme, UBCH5B, C85S UBCH5B-Ub oxyester hydrolysis, and auto-ubiquitination assays were used to address the role of the non-RING elements in E2-E3 interaction and in the enzymatic activity of the RING. The results support that the non-RING elements including the NRGA and TIER motifs are required for E2-E3 recognition and interaction and for efficient auto-ubiquitination. Furthermore, while Arkadia isoform-2 and its close homologue Arkadia 2C are known to interact with free ubiquitin, the results here showed that Arkadia isoform-1 does not interact with free ubiquitin.

## 1. Introduction

Ubiquitination is a post-translational modification that targets proteins for proteasomal degradation [1]. Moreover, ubiquitination regulates protein functions by altering their stability, activity, localization, and protein–protein interaction network [2,3]. In general, ubiquitination involves the sequential action of E1 activating enzyme, E2 ubiquitin conjugation enzymes, and E3 ubiquitin ligases. E1 activates and transfers ubiquitin to E2’s catalytic cysteine to produce an E2~Ub thioester intermediate. E3 ligases, which bind the substrate, interact with the conjugated ubiquitin-E2 enzyme and promote the transfer of ubiquitin from E2 to a nucleophilic residue, which is usually a lysine side chain [4,5]. E3 ligases fall into two major classes, HECT (homologues to the E6-AP carboxyl terminus) and RBR (RING-in-between-RING) ligases, which covalently bind ubiquitin before its transfer to the substrate and RING (really interesting new gene) and RING-like [PHD (plant homeodomain) and U-box] ligases which do not bind ubiquitin and stimulate direct ubiquitin transfer from E2 to the substrate [5,6,7,8].

RING and RING-like E3 ligases represent the large majority of E3 ligases in mammalian cells (over 600) [7,8]. RINGs coordinate two zinc ions in a cross-brace arrangement, which along with an *α*-helix create the binding platform for E2 enzymes [8]. The RING-E2 binding is generally of low affinity in vitro, and in many cases additional interactions are required to form a productive ubiquitination complex and to promote ubiquitin transfer to a substrate [7]. It has become apparent that non-covalent binding of ubiquitin to the “backside” of a subclass of E2 enzymes also facilitates ubiquitination [9,10,11]. It is also known that residues of the E3 enzymes outside the RING domain (non-RING elements) bind their cognate E2 enzymes on secondary sites, away from the canonical RING-E2 interface [7,12,13,14,15,16]. The majority of non-RING elements compete with ubiquitin for the “backside” binding on E2 enzymes [15] leading to either enhancement of polyubiquitination [7,12] or to monoubiquitination [13,16]. The identity and the role of non-RING elements of E3 ligases on E2 recruitment and selection, is therefore important in understanding E3 ligase functions.

E3 ubiquitin ligase Arkadia enhances transforming growth factor-*β* (TGF-*β*)-SMAD2/3 signaling by targeting for ubiquitin proteasomal system (UPS) degradation of the negative regulators SMAD7, a receptor inhibitor, and the nuclear corepressors SKI and its close homologue SKIL (SNON) when these interact with the receptor-activated effectors pSMAD2/3 [17,18,19]. Moreover, Arkadia has been also shown to enhance TGF-*β* signaling by ubiquitinating and degrading pSMAD2/3, which most likely are repressed and bound to the corepressors SKI/SNON [19]. The role of Arkadia in cancer is dual like that of the TGF-*β* pathway, i.e., it promotes tumor suppression in normal cells and metastasis in tumor cells [20,21,22] and for this, it is a candidate drug target against metastasis.

The three-dimensional (3D) structure determination of Arkadia’s RING-H2 domain consisting of 68 aa (aa 927–994, PDB code: 2KIZ), has revealed that it adopts the typical *ββα* fold of RING domains [23]. Moreover, nuclear magnetic resonance (NMR) interaction studies of Arkadia’s RING domain with the E2 enzyme UBCH5B allowed the identification of the residues that participate in the E3-E2 interaction [23,24]. Furthermore, the sequence segment to the N-terminal of the RING-H2 up to aa 889 and consisting of a total 119 aa includes the evolutionary conserved motifs asparagine-arginine-glycine-alanine (NRGA) and threonine-isoleucine-glutamine-arginine (TIER) and a putative nuclear localization signal (NLS) [19,22]. Both motifs have been shown to be required for Arkadia’s interaction with pSMAD2/3 in vivo [19].

Arkadia has been also shown to be required for efficient repair of UV-induced DNA lesions. Arkadia interacts with the UBCH13-MMS2 complex to mediate ubiquitination of SUMOylated substrates such as XPC (Xeroderma Pigmentosum C), a central UV-induced DNA damage recognition factor [25,26]. UBCH13-MMS2 specifically catalyzes the formation of unanchored K63-linked ubiquitin chains or K63-linked chains to substrates, including XPC, indicating a critical role for K63-linked chains in DNA repair mechanism [25,27]. Arkadia’s ability to promote UBCH13-MMS2-dependent ubiquitination of SUMOylated substrates requires its RING domain integrity, whereas mutations of the SUMO-interacting motifs (SIMs) do not affect its E3 ligase activity [25].

Arkadia has four isoforms produced by alternative splicing. Arkadia’s isoform 1 (canonical sequence) and isoform 3 contain a small insertion after the NLS and before the RING (aa 914–921). Wright et al. [28] demonstrated that the isoform 2 of Arkadia that lacks this insertion interacts with Ub. The authors proposed a mechanism where Arkadia RING-Ub complex stabilizes the UBCH5B~Ub conjugate and enhances ubiquitination [28].

Therefore, what it is described herein, is the in-depth investigation of the functional role of Arkadia’s conserved non-RING polypeptide segments (50 aa to the N-terminal of the RING), which are vital for the efficient recruitment of the E2 enzyme and Arkadia’s E3 enzymatic activity.

## 2. Results and Discussion

### 2.1. The RING Domain of Arkadia Is Not Sufficient for Enzymatic Activity

**Figure 1:**
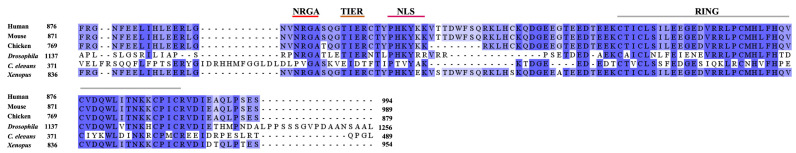
Alignment of C-terminus sequence of Arkadia from different organisms produced by Jalview and colored according to their similarities in amino acids sequence (>80% dark blue, >60% medium dark blue, >40% light blue, ≤40% no color). The NRGA, TIER, NLS, and RING domain are highlighted in orange, brown, purple, and grey lines, respectively.

**Figure 2:**
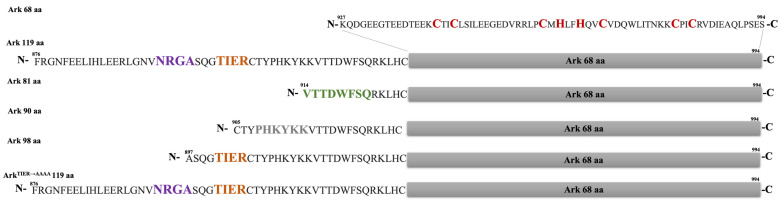
C-terminal constructs of Arkadia E3 ligase. Zinc binding amino acids are colored red, NRGA segment is colored magenta, TIER segment is colored orange, unique for isoform 1 sequence (Val914-Q921) is colored green, and the NLS is colored grey.

**Figure 3:**
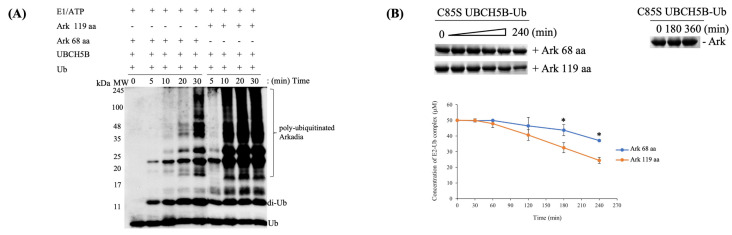
(**A**) In vitro auto-ubiquitination assays of Ark 68 aa and Ark 119 aa polypeptides. Control reactions were conducted without (addition of) Arkadia. All auto-ubiquitination experiments were performed in triplicate. Di-Ub: Ubiquitin dimers. (**B**) Top, oxyester hydrolysis assays showing the disappearance of C85S UBCH5B-Ub in the presence of Ark 68 aa and Ark 119 aa polypeptides, over time. Control reaction demonstrating Oxyester complex’s stability over time. Bottom, densitometry quantification from gels (bars indicate range of experimental duplicates). * Indicates statistically significant difference (*p* value ≤ 0.05).

Arkadia’s activity as an E3 ubiquitin ligase depends on the integrity of its RING-H2 domain [19,22]. However, a highly conserved region at its C-terminus (Figure 1), which includes the NRGA and TIER motifs (894–897 and 901–904 aa, respectively) and a nuclear localization signal (NLS) (908–914 aa), have been shown to be important in cell functional assays [19,22]. Here, we address the role of these motifs in the enzymatic activity of Arkadia using in vitro ubiquitination assays and various Arkadia polypeptides (Figure 2). The results showed that Arkadia 68 aa containing only the RING domain was not sufficient for auto-ubiquitination (Figure 3A). On the contrary, Arkadia 119 aa containing the NRGA, TIER, NLS, and RING-H2 domain was functional. These results suggest that the non-RING elements are essential for enzymatic activity of Arkadia.

To gain a profound insight on the effect of the elements outside the RING domain on Arkadia’s function, oxyester hydrolysis assays using Ark 68 aa and Ark 119 aa polypeptides, were performed. This showed faster disappearance of UBCH5B-Ub complex in the presence of Ark 119 aa polypeptide, compared to Ark 68 aa, suggesting the necessity of the non-RING elements for UBCH5B-Ub conjugate stabilization and Ub release (Figure 3B).

Several studies show that additional elements to the RING domain are required for enzymatic activity, including the interaction between RNF125 and E2 enzyme UBCH5A [29]. The E3 ubiquitin ligase gp78 includes a high affinity binding site for its cognate E2 enzyme, UBE2G2. This UBE2G2 binding region (G2BR) binds UBE2G2 through an extended interface distinct from binding sites for RING and E1 enzyme and increases affinity of gp78 RING-UBE2G2, which is reflected in enhanced ubiquitination [12]. Chaugule et al. 2020 [30] showed by using isothermal titration calorimetry (ITC) that non-RING elements of FANCL E3 ligase increased the affinity for its cognate E2 enzyme UBE2T by two-fold. Moreover, in vitro ubiquitination assays revealed that the presence of non-RING elements enhanced monoubiquitination of FAND2 substrate [30]. In contrast, binding of the structurally unique UBCH5B binding region (U5BR) of AO7/RNF25 E3 ligase to the UBCH5B increases RING-UBCH5B affinity but decreases the rate of ubiquitination favoring monoubiquitination [16]. Using NMR interaction studies and ubiquitination assays, Hibbert et al. 2011 revealed that C-terminal RAD6 binding domain (R6BD) of E3 ligase RAD18 increases RAD6-RING RAD18 affinity and favors monoubiquitination [13]. Interestingly, G2BR, U5BR, and R6BD are mostly characterized by a prominent *α*-helix that crosses the “backside” surface of UBCH5B and enhances interaction with their cognate E2 enzymes (Figure S1). Comparably, Arkadia’s non-RING elements, according to the AlphaFold program, consists of three *α*-helixes (F880-L890, Q899-C905, T916-K923) (Figure S2), which may contact the “backside” of UBCH5B.

**Figure 4:**
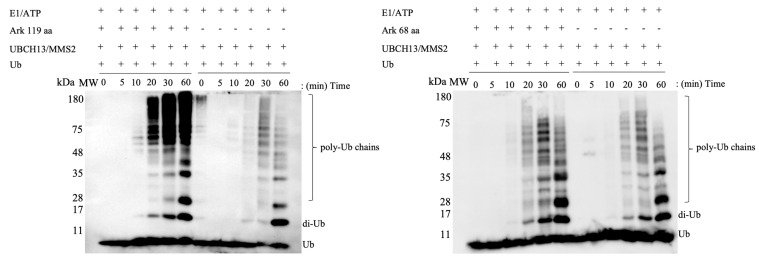
In vitro ubiquitination assays showing the formation of unanchored K63-linked chains after incubation of either Ark 68 aa or Ark 119 aa polypeptides using UBCH13/MMS2 E2 enzymes complex. All ubiquitination experiments were performed three times each.

Ubiquitination assays using Ark 68 aa and Ark 119 aa with the complex of the two E2 enzymes UBCH13-MMS2 revealed that the Ark 119 polypeptide enhances the formation of unanchored K63-linked poly-ubiquitin chains beyond those observed for RING or UBCH13/MMS2 alone (Figure 4). The above results indicate that non-RING elements are significant not only for UBCH5B, but also for UBCH13-MMS2. UBCH5B~Ub and UBCH13~Ub conjugates adopt either an “open” (few non-covalent interactions between E2 and Ub) or a “closed” (multiple contacts between E2 and Ub) conformation and are stabilized to the “closed” conformation by E3 RING ligases [31,32]. Thus, similarly to the E3 ubiquitin ligases RNF25, RNF125, gp78, and RAD18, Arkadia utilizes non-RING elements, whose interaction with E2 enzymes enhances RING-E2 affinity and affects the rate of ubiquitination.

### 2.2. Non-RING Elements Present in Ark 119 aa Polypeptide Enhance E2-E3 Interaction

**Figure 5:**
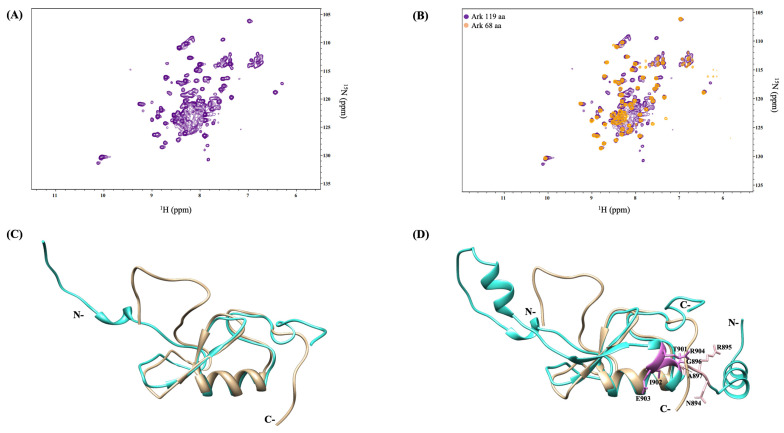
(**A**) ^1^H-^15^N HSQC spectrum of Arkadia 119 aa polypeptide. (**B**) Superposition of the ^1^H ^15^N HSQC spectrum of Arkadia 68 aa with that of Arkadia 119 aa polypeptide. Most of the well dispersed resonances in the ^1^H ^15^N HSQC spectrum of Arkadia 119 aa overlap with those of the Arkadia 68 aa. (**C**) Overlay of the mean NMR model (colored in tan) and AlphaFold predicted structure (colored in turquoise) of Arkadia 68 aa. The superposition is almost perfect except for the disordered loop regions. (**D**) Overlay of the mean NMR model (colored in tan) and AlphaFold predicted structure (colored in turquoise) of Arkadia 119 aa. NRGA and TIER motifs are highlighted with pink and orchid, respectively.

Acquisition of ^1^H-^15^N HSQC NMR spectrum of Arkadia 119 aa polypeptide indicates the coexistence of well-dispersed resonances (characteristic of the RING domain resonances), with broad, non-dispersed, and overlapped signals, typical for non-structured peptide segments (Figure 5A). Signal overlap and spectral crowding are mostly observed in the ppm range 7.9–8.6 ppm (in ^1^H dimension) and 120–126 ppm (in ^15^N dimension), suggesting the intrinsic high flexibility of the non-RING resonances, at the N-terminus. Together, the above data show that the polypeptide segment proceeding the RING is probably disordered and the resonance features prevents the assignment of its residues (Figure 5B). Using the AphaFold database from DeepMind [33], which predicts protein structure from amino acid sequence, a 3D model of full-length Arkadia was generated. Comparison of the 3D structure of Arkadia’s RING domain determined by NMR spectroscopy (PDB code: 2KIZ) with the 3D structure provided by AlphaFold program indicated significant convergence between the NMR and the predicted Arkadia 68 aa, when the 68-residue polypeptide segment compared with the corresponding segment of the modeled Arkadia 119 aa (Figure 5C,D).

**Figure 6:**
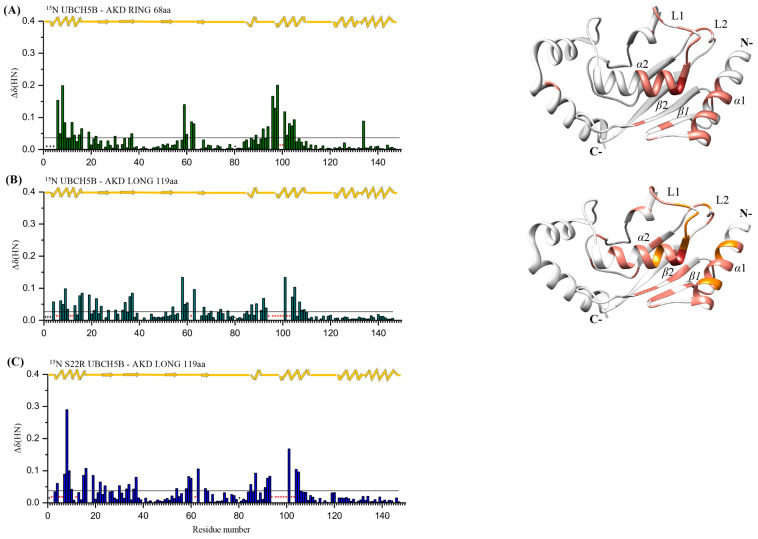
Bar graphs of UBCH5B’s CSPs and UBCH5B mapping (PDB code: 2ESK) after addition of: (**A**) Ark 68 aa and (**B**) Ark 119 aa. The residues that disappeared during the interaction are colored red, while the residues that exhibited fast exchange interaction are colored coral. Residues that change from fast to intermediate exchange due to the stronger binding with Ark 119 aa are colored orange. (**C**) Bar graphs of S22R UBCH5B’s CSPs after addition of Arkadia 119 aa. *: Represents disappeared residues, +: represents residues with no information.

The interaction of E2 enzyme UBCH5B with the RING domain of Arkadia via NMR spectroscopy, has been previously studied [26] and shows that Arkadia’s RING domain binds UBCH5B using its two Zn^2+^-coordinating loops and the intervening central *α*-helix. The interaction involves the UBCH5B *α*1-helix and L1 and L2 loops [23]. The studies described herein, on the interaction of ^15^N labeled UBCH5B [34] with Ark 119 aa, containing non-RING elements, revealed that most of the resonances exhibited either fast or intermediate exchange in NMR time-scale (Figure 6B). Furthermore, addition of the unlabeled Ark 119 aa into a solution of ^15^N-labeled UBCH5B produced chemical shift perturbations (CSPs) of the residues corresponding to helixes *α*1, *α*2 as well as loops L1 and L2 and *β*2 strand and extracted CSPs are depicted in Figure 6 The amide resonances of UBCH5B Arg5, Ile6, Asn11, Asp12, Phe62, Ser94, Ala96, Leu97, Thr98, Ile99, Ser100, Val102, and Leu103 broadened beyond detection and remained undetectable suggesting interaction. Comparison of the CSPs in UBCH5B caused by the interaction with Ark 68 aa (Figure 6A) and Ark 119 aa (Figure 6B) shows that more and larger CSPs occur with Ark 119 aa at the same molar ratio of 1:2 (Figure S3). The above findings show that the RING domain binds UBCH5B, but this interaction is enhanced by the presence of the N-terminal non-RING region (876–927 aa).

**Figure 7:**
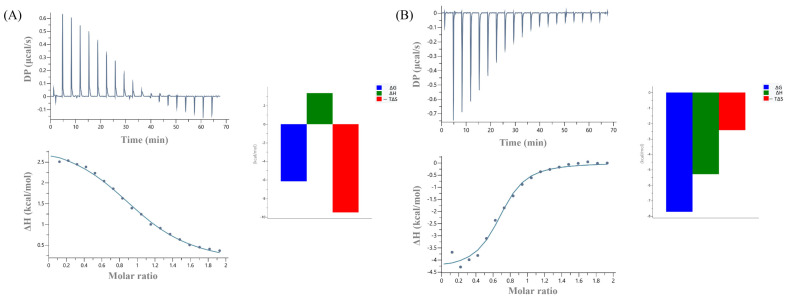
ITC data for the titration of UBCH5B into (**A**) Arkadia 68 aa and (**B**) Arkadia 119 aa.

To further study the impact of the non-RING elements in the recruitment of the E2, ITC experiments were carried out. According to these data, binding affinity of the Ark RING 68 aa with UBCH5B was found 32 μM, whereas the one of the Ark 119 aa with UBCH5B was 2.7 μM (Table 1). Notably, Ark 68 aa-UBCH5B interaction was endothermic and entropy-driven, whereas Ark 119 aa-UBCH5B interaction was exothermic and enthalpy- and entropy-driven (Figure 7).

To test whether Arkadia’s non-RING elements binding to the “backside” surface of UBCH5B implicates Serine 22 (S22), the S22R UBCH5B mutant was prepared and used for NMR interaction studies. Mutation of UBCH5 Ser22 to an Arg, eliminates Ub’s noncovalent backside binding [9,10]. NMR interaction studies of the ^15^N-labeled S22R UBCH5B mutant with the unlabeled Ark 119 aa and subsequent CSPs analysis revealed that the S22R UBCH5B-Ark 119 aa CSPs graph was identical to the wt UBCH5B-Ark 119 aa CSPs graph (Figure 6B,C). These results suggest that Ser22’s substitution by a long-chain, basic, amino acid such as arginine, has no effect on Ark 119 aa polypeptide interaction with UBCH5B “backside” region.

### 2.3. Arkadia Isoform 1 Does Not Bind Ubiquitin

**Figure 8:**
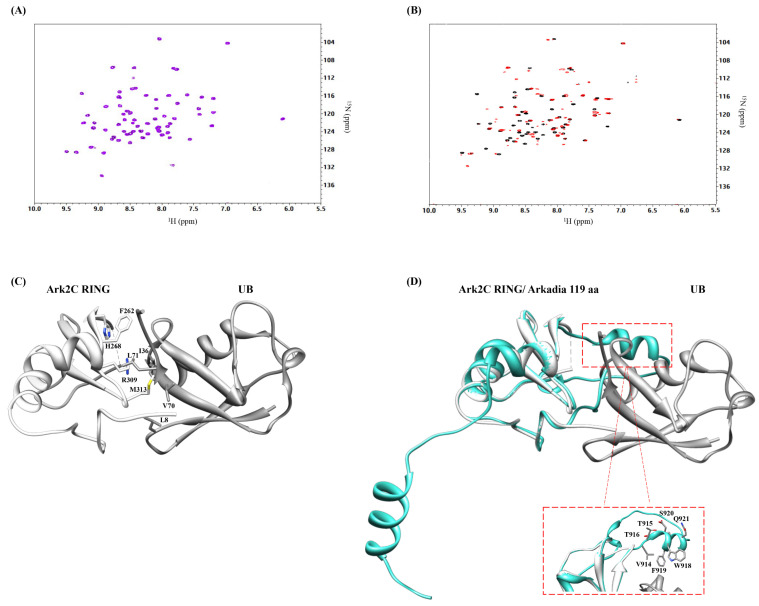
^1^H-^15^N HSQC spectrum of Ub (black) after addition of either (**A**) Arkadia 119 aa isoform 1 (magenta) or (**B**) Arkadia isoform 2 (red). (**C**) Representation of Arkadia 2C (Ark2C)-Ub complex. (**D**) Arkadia 119 aa AlphaFold predicted structure (cyan) matched onto the Ark2C bound protein (white) structure using the MatchMaker tool of UCSF Chimera.

Previous studies using constructs of Arkadia isoform 2 and its close homologue Arkadia 2C, comprising the ~100 C-terminal residues, showed that free ubiquitin binds the *β*3-sheet, interacts with the E2-conjugated ubiquitin. This ubiquitin–ubiquitin interaction stabilizes the E2~Ub conjugate and enhances ubiquitin transfer [28]. NMR titration of Ark 119 aa (isoform 1) with free ^15^N-labeled ubiquitin showed no interaction between these two proteins (Figure 8A). In contrast, the NMR studies of Arkadia isoform 2 (876–986 aa) with ubiquitin confirm their interaction (Figure 8B).

**Figure 9:**
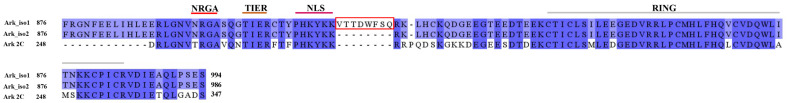
Alignment of Arkadia isoform 1, Arkadia isoform 2 and Arkadia 2C produced by Jalview and colored according to their similarities in amino acids sequence (dark blue for conserved residues, medium dark blue for conserved type of residues and no color for non-conserved residues). Red box: amino acids which do not appear in Arkadia isoform 2 and Arkadia 2C.

Alignment of Arkadia isoform 1, isoform 2, and Arkadia 2C revealed a segment of amino acids (Val914-Q921) unique for Arkadia isoform 1 (Figure 9). Crystal structure of Arkadia 2C RING in complex with UBCH5B~Ub (PDB code: 5D0K) revealed that Arkadia’s 2C ubiquitin binding surface includes the *β*3-strand and a disorder region (Figure 8C and Figure S5). Using the UCSF Chimera program [35] and the MatchMaker extension Ark 119 aa model, was superimposed with Arkadia 2C RING-Ub complex (Figure 8D). From the superposition it seems that these seven amino acids, which are a “signature” segment missing from Arkadia isoform 2 and Ark 2C, affected the interaction surface of Arkadia isoform 1 causing the loss of Arkadia’s Ub binding. However, auto-ubiquitination assays indicated that the loss of the ubiquitin binding does not affect ubiquitination function of Arkadia isoform 1.

### 2.4. Screening Arkadia’s Non-RING Sequence That Is Crucial for E2 Interaction and E3 Ligation Activity

To determine the role of additional non-RING elements of Arkadia the Ark 81 aa and Ark 90 aa polypeptides were used (Figure 2). Both polypeptides lack NRGA and TIER motifs but Ark 81 aa contains the unique segment (Val914-Q921 aa) of isoform 1, while Ark 90 aa is longer and contains the NLS domain.

**Figure 10:**
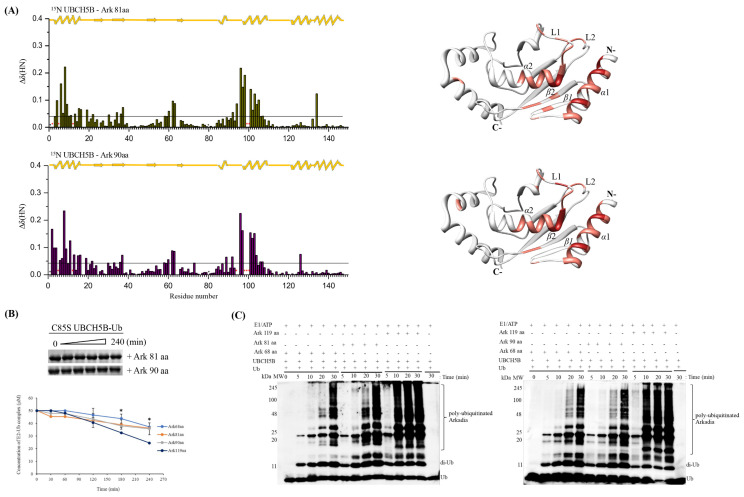
(**A**) Bar graphs of UBCH5B’s CSPs after addition of either Ark 81 aa or Ark 90 aa. *: Represents disappeared residues, +: represents residues with no information. (**B**) Top, oxyester hydrolysis assays showing the disappearance of C85S UBCH5B-Ub in the presence of Ark 68 aa, Ark 81 aa, Ark 90 aa, and Ark 119 aa polypeptides, over time. Bottom, densitometry quantification from gels (bars indicate range of experimental duplicates). * Indicates statistically significant difference (*p* value ≤ 0.05). (**C**) In vitro auto-ubiquitination assays of those polypeptides. Control reactions were conducted without addition of Arkadia. All auto-ubiquitination experiments were performed in triplicate.

The ^1^H-^15^N HSQC spectra of these polypeptides were similar to Ark 119 aa polypeptide, comprised of the structured RING domain and disordered segments (Figure S6). We hypothesized that the unique sequence of Arkadia isoform 1 binds to the secondary sites of UBCH5B and enhances E2-E3 interaction. NMR interaction studies of ^15^N UBCH5B with Arkadia 81 aa and CSPs analysis showed that most of the resonances exhibited fast exchange behavior, suggesting a weak interaction. The amide resonances Ile6, Asp12, Ile99, and Ser100 of UBCH5B disappeared and remained undetectable. UBCH5B CSPs after addition of Arkadia 81 aa closely resemble CSPs obtained with Arkadia’s RING (Figure 10A). Moreover, hydrolysis and ubiquitination assays showed that functional properties of Ark 81 aa most closely resemble that of Ark 68 aa (Figure 10B,C).

**Figure 11:**
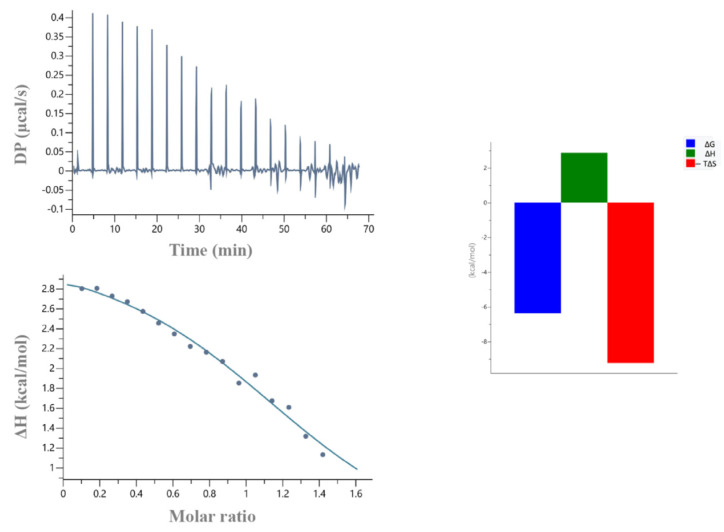
ITC data for the titration of UBCH5B into Arkadia 90 aa.

Then, we investigated with the Ark 90 aa whether the NLS putative domain outside the RING is implicated in E2 interaction. NMR interaction studies were conducted using ^15^N UBCH5B and the results revealed that Ark 90 aa polypeptide caused to the UBCH5B the same chemical shift changes, as those observed for Arkadia 81 aa polypeptide (Figure 10A). The amide resonances Arg5, Asp12, Ser94, Thr98, Ile99, and Ser100 of UBCH5B broadened beyond detection and were hard to be identified. ITC experiments using Arkadia 90 aa and UBCH5B showed that their dissociation constant was 23 μM, which is similar with the one found for the Ark 68 aa-UBCH5B pair (32 μM) (Figure 11) and ~10 times lower than the Ark 119 aa-UBCH5B-interacting protein pair. Additionally, hydrolysis and ubiquitination assays showed that Ark 90 aa exhibited similar activity with the Ark RING 68 aa and Ark 81 aa (Figure 10B,C). All the above-mentioned results indicate that the amino acids 905–926 to the N-terminal of the RING do not enhance RING binding to E2 and ubiquitination activity.

### 2.5. NRGA and TIER Segments Implication in E2 Interaction and Ubiquitination

**Figure 12:**
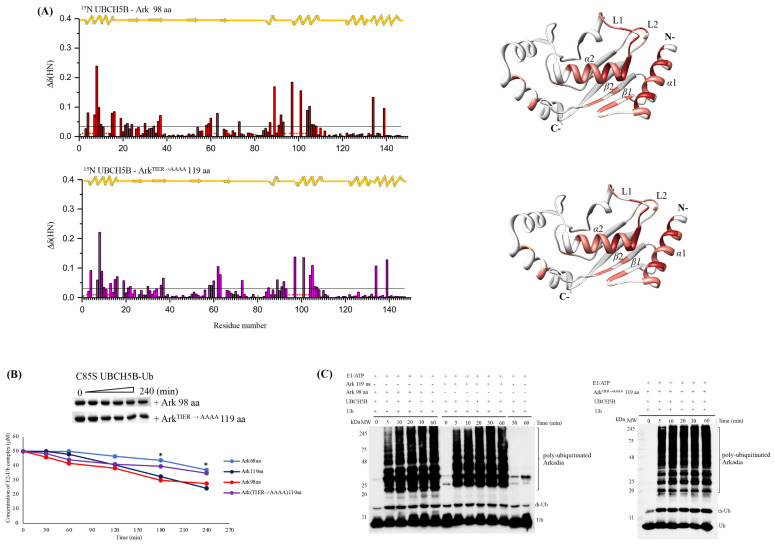
(**A**) Bar graphs of UBCH5B’s CSPs after addition of Arkadia ΔRING (98 aa) or Arkadia^TIER→AAAA^ (119 aa) polypeptides. *: Represents disappeared residues, +: represents residues with no information. (**B**) Top, oxyester hydrolysis assays showing the disappearance of C85S UBCH5B-Ub in the presence of Ark 68 aa, Ark 98 aa, Ark 119 aa, and Ark^TIER→AAAA^ 119 aa polypeptides, over time. Bottom, densitometry quantification from gels (bars indicate range of experimental duplicates). * Indicates statistically significant difference (*p* value ≤ 0.05). (**C**) In vitro auto-ubiquitination assays of Ark 98 aa and Ark^TIER→AAAA^ (119 aa) polypeptides. All auto-ubiquitination experiments were performed in triplicate.

To determine whether the Arkadia NRGA and TIER segments have a role in enhancing E2-E3 interaction and ubiquitination activity, two new Ark polypeptides were generated, ΔNRG (Ark 98 aa) and the TIER* (119 aa) containing a AAAA replacement (Ark^TIER→AAAA^ 119 aa) (Figure 2). NMR titration experiments using isotopically labelled UBCH5B with Ark 98 aa or Ark^TIER→AAAA^ 119 aa peptides, revealed that the UBCH5B interaction surface involves the same amino acids as the Ark 119 aa polypeptide, which are in *α*1 and *α*2 helixes, L1 and L2 loops, and *β*2 strand (Figure 12A). Interestingly, Phe62, located in L1 loop of UBCH5B, exhibits different behavior during titration in these two polypeptides. Specifically, Phe62 NH peak in ^15^N-HSQC, disappeared and remained undetectable after addition of Ark 98 aa, in a similar way as that observed for Ark 119 aa. Phe62 is conserved among UBC4/UBC5 E2 enzymes subfamilies and it is critical for E3 binding [36]. Hydrolysis assays, using Ark 98 aa and Ark^TIER→AAAA^ 119 aa polypeptides revealed decrease of UBCH5B-Ub conjugate hydrolysis rate for Ark^TIER→AAAA^ 119 aa, compared to Ark 98 aa and Ark 119 aa (Figure 12B) suggesting that the TIER segment is implicated in the stabilization of UBCH5B~Ub conjugate in the “closed” conformation. On the contrary, the ubiquitination assay showed that the NRG deletion and TIER replacement has not affected the enzymatic function of Arkadia most likely because the condition of this assay exhibits low detection sensitivity (Figure 12C).

Together, these experiments show that the NRGA and TIER motifs enhance the E2-E3 interaction and are required for the enzymatic ligase activity of the RING, while the unique segment of isoform 1 and NLS most likely are not involved.

## 3. Materials and Methods

### 3.1. Construct Design

In an attempt to screen and determine the minimal length of Arkadia isoform 1 that is sufficient for its E3 ligase function, the Ark RING 68aa, Ark 119 aa, Ark 81 aa, Ark 90 aa, Ark 98 aa, and Ark^TIER→AAAA^ 119 aa constructs were designed. Determining the construct boundaries was based on AlphaFold protein structure database (Figure S2), on biochemical data supporting that the C-terminal 100 amino acids are required for Arkadia’s activity [19,22] and on sequence alignment with Arkadia isoform 2 and Arkadia 2C. The constructs are named according to their length in amino acids.

### 3.2. Constructs, Protein Expression and Purification

Human Arkadia (RNF111) constructs were cloned into pGEX-4T-1 in frame with a N-terminal GST-tag. Arkadia polypeptides used in this study include residues 927–994 (Ark RING 68aa), 876–994 (Ark 119 aa), 914–994 (Ark 81 aa), 905–994 (Ark 90 aa), and 897–994 (Ark 98 aa). The Arkadia 119 aa polypeptide bearing the TIER segment mutated to alanine was synthesized with codon optimization for expression in *Escherichia coli* cells (Genscript Biotech). The *E. coli* cells were grown at 37 °C in minimal medium supplemented with ^15^NH_4_Cl and 1/1000 ^15^N BioExpress^®^ (Cambridge Isotope Laboratories, CIL, Tewksbury, MA, USA). When the optical density (OD_600_) reached 0.6–0.9, induction was carried out using 1 mM isopropyl β-d-1-thiogalactopyranoside (IPTG). Cells were harvested after 4 or 5 h from induction and were lysed using sonication in phosphate buffered saline (PBS) pH 7.4 ± 0.2 lysis buffer supplemented with protease inhibitors cocktail (Sigma-Aldrich, St. Louis, MO, USA) and DNase I (GeneOn, Ludwigshafen, Germany). GST-tagged proteins were purified by affinity chromatography using GST-trap columns (GE Healthcare, Chicago, IL, USA) and PBS. The GST-tag was cleaved after overnight incubation at room temperature with thrombin (Merck Millipore, Burlington, MA, USA). Arkadia polypeptides were further purified by size exclusion chromatography with Superdex^®^ 75 10/300 GL column (GE Healthcare, Chicago, IL, USA) that previously had been equilibrated with 50 mM K_2_HPO_4_ and 50 mM KH_2_PO_4_, pH 7. The purified proteins were frozen and stored at −80 °C until use. UBCH5B, UBCH13, and MMS2 (UBE2V2) proteins were expressed using Rosetta™ 2(DE3) Singles™ cells (Novagen, Darmstadt, Germany). The bacterial cells were grown in minimal medium supplemented with ^15^NH_4_Cl and 1/1000 ^15^N BioExpress^®^ (Cambridge Isotope Laboratories, CIL, Tewksbury, MA, USA). After OD_600_ reached values of 0.6–0.9, IPTG was added at a final concentration of 1 mM and the temperature was lowered to 18 °C. The cells were harvested after 16 h and were lysed by sonication. The proteins were purified by metal ion affinity chromatography using increasing concentrations of imidazole buffers (10, 20, 40 mM imidazole and 20 mM Na_2_HPO_4_, 500 mM NaCl pH 8). The His_6_-tag was removed after overnight incubation at room temperature with thrombin (Merck Millipore, Burlington, MA, USA) and the proteins were eluted at 10 mM imidazole. This was followed by further purification with size exclusion chromatography by Superdex^®^ 75 10/300 GL column equilibrated in 50 mM K_2_HPO_4_ and 50 mM KH_2_PO_4_ pH 7. The S22R mutation was introduced into UBCH5B using PCR-based site-directed mutagenesis and the protein was expressed and purified as described for the wild type (wt) protein. Untagged ubiquitin was purified by heating at 85 °C for 15 min and centrifugation at 13,000 rpm for 20 min. Ubiquitin was further purified by size exclusion chromatography as described for Arkadia and UBCH5B.

### 3.3. Isothermal Titration Calorimetry (ITC)

ITC experiments were conducted using a Microcal PEAQ ITC (Malvern, United Kingdom) at 25 °C [37]. Typically, 75–200 μM of Arkadia polypeptides were loaded into cell, and 0.75–2 mM UBCH5B was delivered by a series of 2 μL injections from the syringe with constant stirring at 750 rpm. Each of the injections was separated by 210 s intervals to allow the system to reach baseline. Raw data were processed in Microcal PEAQ ITC analysis software (Malvern, United Kingdom) using a single site-binding model.

### 3.4. Auto-Ubiquitination Assays

For the in vitro ubiquitination assays, the reactions were performed as described in Birkou et al. 2022 [38]. Briefly, to a total volume of 20 μL, 20 mM Tris-HCl, 50 mM NaCl pH 7.5, 5 mM ATP, 2 mM MgCl_2_, 2 mM DTT, 1 μM E1, 5 μM UBCH5B or 10 μM UBCH13/MMS2, 15 μM Arkadia, and 100–150 μM Ub were added. Reactions were incubated at 37 °C and samples were collected at specific time points. The reactions were stopped with SDS/DTT loading buffer and the reaction products were separated by 15% SDS-PAGE followed by Western blotting using anti-Ub (Santa Cruz Biotechnology, SCB, Dallas, TX, USA) and visualized using ChemiDoc Imaging System (Biorad, Hercules, CA, USA).

### 3.5. Synthesis of E2-Ub Conjugate

For experiments using oxyester-linked UBCH5B-ubiquitin complexes, an active site Cys-to-Ser mutation (C85S UBCH5B) was performed. The resulting bond was only one atom different from the wt thioester and significantly more stable. A successful conjugation reaction was typically accomplished by mixing 1 µM E1, 150–200 µM E2, 500–600 µM ubiquitin, cycling buffer (50 mM Tris-HCl, 150 mM NaCl, pH 7), 10 mM creatine phosphate (Sigma-Aldrich), ~0.6 units mL^−1^ creatine kinase (Sigma-Aldrich), 5 mM MgCl_2_, and 5 mM ATP. Conjugation reactions were incubated at 37 °C for 16–20 h, and the reaction was monitored using SDS–PAGE. Oxyester linked UBCH5B-ubiquitin was purified from E1 and unreacted proteins using a Superdex^®^ 75 10/300 GL size-exclusion chromatography column (GE Healthcare) equilibrated in 50 mM K_2_HPO_4_ and 50 mM KH_2_PO_4_ pH 7 [24]. The purified C85S UBCH5B–Ub conjugate was collected, flash-frozen in liquid nitrogen and stored at −80 °C.

### 3.6. Oxyester Hydrolysis Assays

UBCH5B-Ub oxyester conjugate hydrolysis assays were conducted by incubating 50 μM of UBCH5B-Ub oxyester complex with 25 μM of Arkadia polypeptides, in 50 mM K_2_HPO_4_, 50 mM KH_2_PO_4_ pH 7, at 25 °C for 30, 60, 120, 180, 240 min. Reactions were terminated by the addition of SDS loading buffer. The protein samples were separated by 15% SDS-PAGE and the gels were stained with Coomassie blue stain [39]. The hydrolysis of UBCH5B-Ub oxyester conjugate was quantified with Image Lab (ChemiDoc Imaging System, Biorad, Hercules, CA, USA).

### 3.7. NMR Titration

Arkadia polypeptides and E2 UBCH5B, titration experiments were monitored by ^1^H–^15^N HSQC spectra of labeled ^15^N UBCH5b after each addition of the unlabeled protein partner. The unlabeled protein was added in eight steps in order to reach the following ratios and saturation of labeled/unlabeled protein: 1:0.25, 1:0.5, 1:0.75, 1:1, 1:1.25, 1:1.5, 1:1.75, 1:2. Combined chemical shift perturbations (CSPs) after binding were calculated using the equation [40,41,42]:

$$\Delta \delta_{ppm}=\sqrt{{\left(\Delta \delta_{HN}\right)}^{2}+{\left(\frac{\Delta \delta_{Ν}}{5}\right)}^{2}}$$

The meaningful CSPs were derived calculating a threshold value for each of the studied interactions. The threshold value was set using the standard deviation [42].

## 4. Conclusions

In this study we report the role of key sequence segments of the Arkadia C-terminal region and show that they are crucial for the Arkadia recognition and interaction with E2 enzyme UBCH5B. Our data provide experimental evidence that Arkadia is an E3 ubiquitin ligase that utilizes non-RING elements to effectively bind the E2 enzyme, and to form a productive E2-E3 complex with poly-ubiquitination capacity. Specifically, our results revealed the key-role of NRGA and TIER domains, which are conserved within the non-RING elements of Arkadia, for E2-E3 interaction and for Arkadia’s enzymatic activity. Moreover, using in vitro ubiquitination assays we showed that non-RING elements of Arkadia are also crucial for UBCH13-MMS2 unanchored chains formation. Interestingly, both UBCH5B and UBCH13 ubiquitin conjugates are stabilized by interacting with RING E3 ligases, suggesting that Arkadia utilizes the same recognition mechanism with these two E2 enzymes. Specifically, we found that the amino acid including either NRG or TIER segments, is sufficient for a productive E2-E3 interaction and poly-ubiquitination activity. Whether the non-RING elements of Arkadia interacts with the “backside” of UBCH5B requires further investigation. Finally, our data revealed that Arkadia isoform 1 consists of an amino acid sequence (Figure 9), which may attenuate the interaction with ubiquitin, as the NMR interaction studies of Arkadia isoform 1 with ubiquitin indicated.

Given the biological role of Arkadia and its impact in cancer progression and metastasis, unveiling the mechanism of its function as an E3 ligase is of significance. Moreover, considering that ubiquitination is a highly complex process, disclosing the structural and biophysical determinants of the E3 Ub ligases, such as Arkadia, that facilitate molecular recognition and ubiquitin transfer, will shed light on the ubiquitination process and open new routes for intervention and potential regulation of this process, with apparent interest for new therapeutic approaches.

## Figures and Tables

**Table 1:** Thermodynamic parameters for the Arkadia 68 aa and Arkadia 119 aa interaction with UBCH5B obtained by ITC at 25 °C.

Protein	K_d_ (μΜ)	N	ΔH (Kcal/mol)	−TΔS (Kcal/mol)	ΔG (Kcal/mol)
Arkadia 68 aa	32	1	3.1	−9.3	−6.14
Arkadia 119 aa	2.7	0.6 *	−4.7	−2.9	−7.61

Each value is the average of at least three independent measurements. Abbreviations: N, stoichiometry; K_d_, dissociation constant; ΔH, ΔS, and ΔG, changes in binding enthalpy, binding entropy, and Gibbs energy, respectively. * N < 1, protein not fully active (probably due to existence of the disorder region. N < 1, usually indicates either protein not fully active or dimer. Supplementary Figure S4).

## Data Availability

The data that support these findings are available upon request from the corresponding author.

## Supplementary Materials

The following supporting information can be downloaded at: https://www.mdpi.com/article/10.3390/ijms231810585/s1.
