# Supplementary material for: Unveiling the Essential Role of Arkadia’s Non-RING Elements in the Ubiquitination Process
Source: Int J Mol Sci. 2022 Sep 13;23(18):10585. doi: 10.3390/ijms231810585 (PMC9501438; doi:10.3390/ijms231810585)
Supplement: Supplementary file 1 [file ijms-23-10585-s001.zip › ijms-1869691-supplementary.pdf]

# SUPPLEMENTARY MATERIALS

# Unveiling the essential role of Arkadia's non-RING elements in the ubiquitination process

Maria Birkou[1], Georgia N. Delegkou[1], Konstantinos D. Marousis[1], Nefeli Fragkaki[1], TamaraToro[1], Vasso Episkopou[2*], Georgios A. Spyroulias[1*]

[1]Department of Pharmacy, University of Patras, Greece
[2]Department of Brain Sciences, Imperial College, London, United Kingdom

Correspondence: vasso.episkopou@imperial.ac.uk; G.A.Spyroulias@upatras.gr

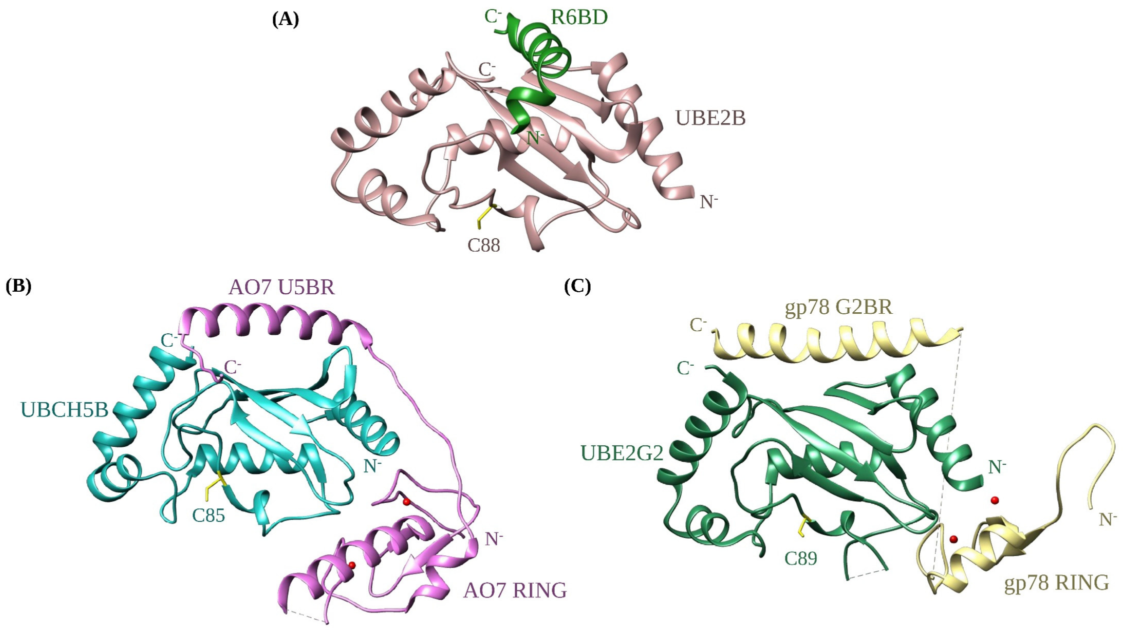


**Figure S1:** Crystal structure of the complexes: A) R6BD (Rad6 binding domain) of Rad18 with UBE2B (PDB code: 2YBF), B) RING-U5BR (UBCH5B binding region) of AO7 with UBCH5B (PDB code: 5D1L) and C) RING-G2BR (Ube2g2 binding region) of gp78 with UBE2G2 (PDB code: 4LAD).

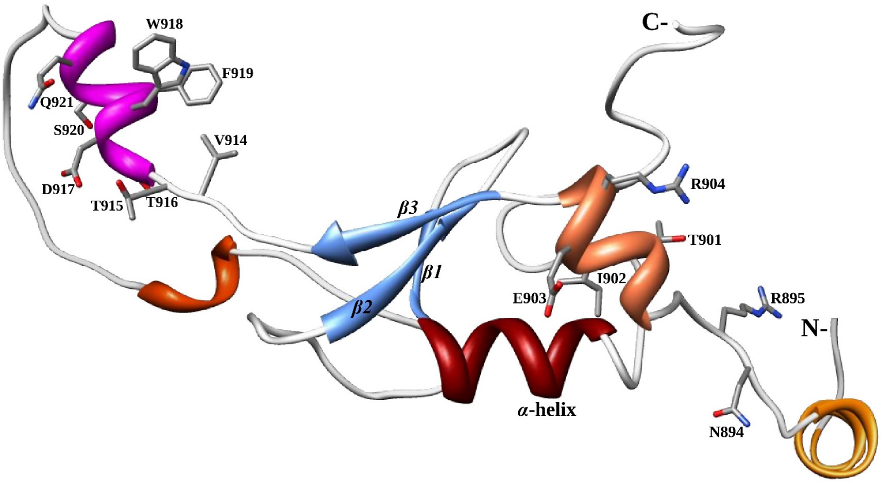


**Figure S2:** 3D structure of Arkadia 119 aa according to AlphaFold protein structure database.

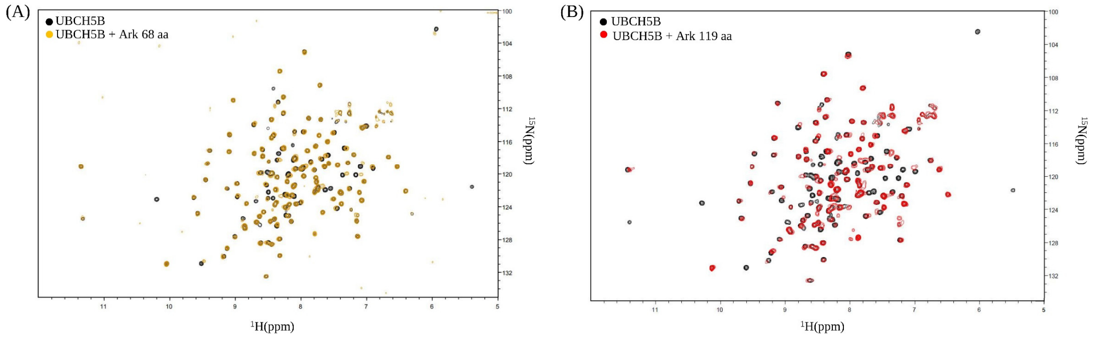


**Figure S3:** Superposition of 2D $^{1}$H-$^{15}$N HSQC spectra of UbcH5b before (black) and after the addition of A) Arkadia 68 aa (yellow) and B) Arkadia 119 aa (red) at a molar ratio 1:2.

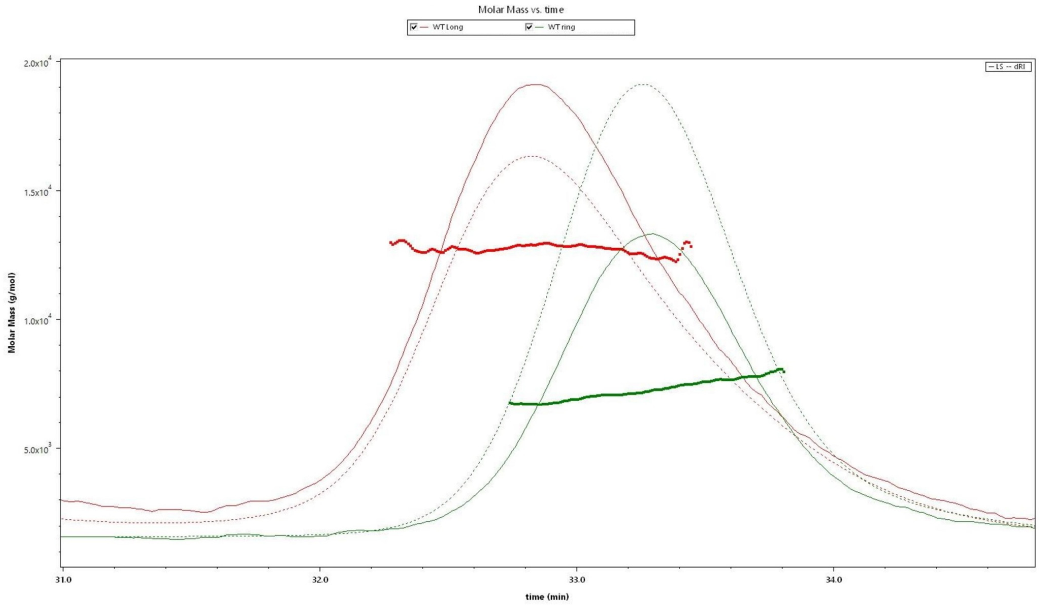


**Figure S4:** Size exclusion chromatography coupled to multiangle laser light scattering (SEC-MALS) was performed using a Superdex™ Increase 200 10/300 GL column attached to a Dawn 8+ MALLS detector (Wyatt Technology). Pure Ark 68 aa and Ark 119 aa proteins (250 μM) buffered by 50 mM $KH_2PO_4$, $K_2HPO_4$, pH 7 were flowed through the system at 0.5 mL/min and data were analyzed with ASTRA 5.3.4 software (Wyatt Technology). Ark 68 aa (RING) monomer (7.3 kDa), Ark 119 aa (LONG) monomer (13 kDa).

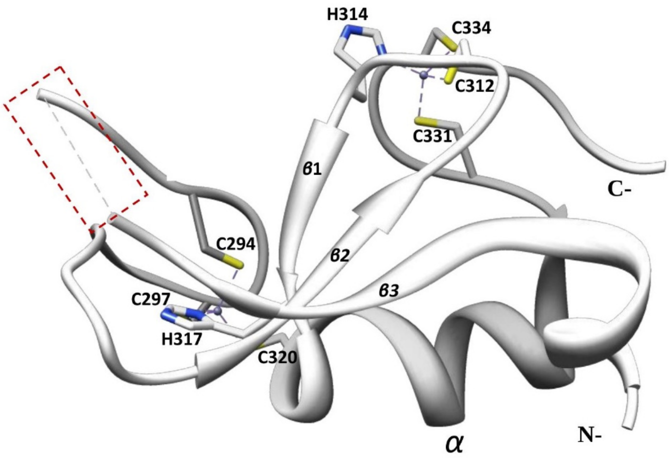


**Figure S5:** Crystal structure of Arkadia 2C 254-346 aa (272-290 aa were deleted) (PDB code: 5D0I).

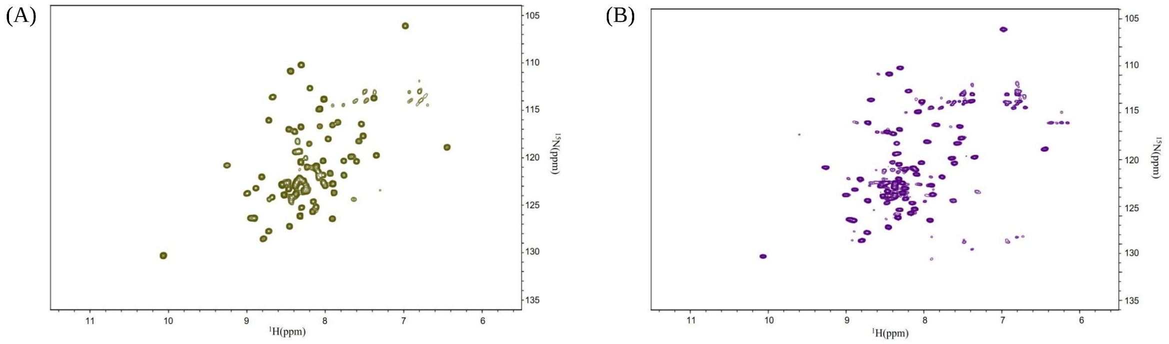


**Figure S6:** $^1$H-$^{15}$N HSQC spectrum of Arkadia A) 81 aa and B) 90 aa polypeptides.
